# Myelinating Glia: Potential Therapeutic Targets in Polyglutamine Spinocerebellar Ataxias

**DOI:** 10.3390/cells12040601

**Published:** 2023-02-13

**Authors:** Alexandra F. Putka, Juan P. Mato, Hayley S. McLoughlin

**Affiliations:** 1 Department of Neurology, University of Michigan, Ann Arbor, MI 48109, USA; 2 Neuroscience Graduate Program, University of Michigan, Ann Arbor, MI 48109, USA

**Keywords:** neurodegeneration, oligodendrocyte, Schwann cell, white matter, peripheral neuropathy

## Abstract

Human studies, in combination with animal and cellular models, support glial cells as both major contributors to neurodegenerative diseases and promising therapeutic targets. Among glial cells, oligodendrocytes and Schwann cells are the myelinating glial cells of the central and peripheral nervous system, respectively. In this review, we discuss the contributions of these central and peripheral myelinating glia to the pathomechanisms of polyglutamine (polyQ) spinocerebellar ataxia (SCA) types 1, 2, 3, 6, 7, and 17. First, we highlight the function of oligodendrocytes in healthy conditions and how they are disrupted in polyQ SCA patients and diseased model systems. We then cover the role of Schwann cells in peripheral nerve function and repair as well as their possible role in peripheral neuropathy in polyQ SCAs. Finally, we discuss potential polyQ SCA therapeutic interventions in myelinating glial.

## 1. Introduction

Spinocerebellar ataxias (SCAs) are a group of autosomal-dominantly inherited neurodegenerative diseases united by common pathological and phenotypic features [1,2]. Pathologically, as the name suggests, spinocerebellar ataxias are characterized by widespread cerebellar degeneration and/or loss of spinocerebellar tracts [1,2,3,4,5]. The second part of the name, ataxia, describes the phenotypic presentation: loss of coordination, usually first observed in a patient’s gait [1,2,3]. Currently, there are at least 47 recognized SCA subtypes [1,6,7]. Of these, six are caused by aberrantly long polyglutamine (polyQ) tracts in disease proteins, encoded by an exonic CAG repeat expansion in the disease gene [8]. These include SCA types 1, 2, 3, 6, 7, and 17 [1,2,3,8]. Importantly, no disease-modifying or curative therapeutics exist for these diseases.

Due to advances in RNA sequencing (RNAseq) and histological staining techniques, myelinating glial cells have emerged as major contributors to neurodegenerative diseases [9,10,11,12,13,14,15,16]. Additionally, research is moving beyond the central nervous system to understand the major debilitating symptom of peripheral neuropathy in polyQ SCAs and other neurodegenerative diseases [16,17,18]. In this review, we discuss the role of myelinating glia, namely, oligodendrocytes in the central nervous system and Schwann cells in the peripheral nervous system, in polyQ SCA pathogenesis. Across human, animal, and cellular models, studies support these cells as major contributors to disease as well as viable therapeutic targets, highlighting the need for additional research on these cell types.

## 2. Central Nervous System Myelinating Glia: Oligodendrocytes

**Figure 1:**
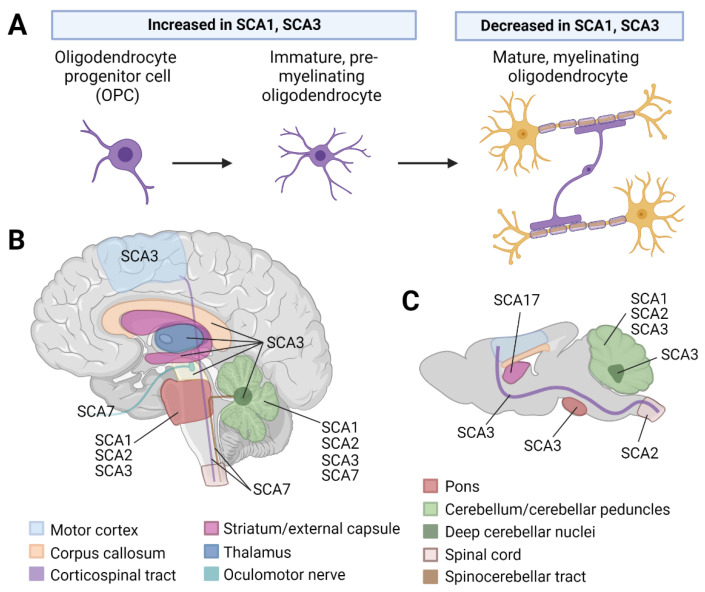
Evidence for white matter and oligodendrocyte abnormalities in polyQ SCA literature. (**A**) There is direct evidence for a perturbation of oligodendrocyte maturation in SCA1 and 3, with an increase in oligodendrocyte progenitor cells (OPCs)/immature oligodendrocytes and a decrease in mature, myelinating oligodendrocytes in disease. (**B**) Diffusion tensor imaging and magnetic resonance spectroscopy studies demonstrate alterations to distinct white matter tracts in SCA1, 2, 3, and 6 patients. (**C**) Mouse models of SCA1, 2, 3, and 17 also support regional white matter alterations or oligodendrocyte involvement in these diseases. Figure created using BioRender.

To understand the contributions of oligodendrocytes in disease, it is essential to first detail the basic biology of oligodendrocytes, including how they mature. To fulfill their chief role of myelination, which promotes efficient conduction of action potentials, oligodendrocytes mature through three stages: oligodendrocyte progenitor cells (OPCs), immature or premyelinating oligodendrocytes, and mature myelinating oligodendrocytes [19,20,21] (Figure 1A). These maturation states are marked by various transcription factors and morphological features [22] (Figure 1A). Myelin is composed of a high lipid to protein content ratio of 75–80% lipids and 20–25% protein. This is in stark contrast to biological membranes, which have an equal composition of lipid and protein [23]. The major components of myelin are galactosylceramide and sulfatide (its sulfated form), which account for 23 and 4% of myelin lipids, respectively [24,25,26]. In addition to myelination, oligodendrocytes metabolically support neurons through the rapid transfer of energy metabolites, including lactate and pyruvate [27]. This is important because neurons are estimated to consume 75–80% of the energy in the brain [28,29], meaning they exert a high burden on the limited energy supply and as such require metabolic support from other cells. In addition to metabolic support, oligodendrocytes provide trophic support to nearby neurons via brain-derived neurotrophic factor and neurotrophin-3 [30]. Finally, oligodendrocytes express regulatory immune factors, such as complement factors, MHC, cytokines, and chemokines [31], implicating crosstalk with microglia as an important component of their normal function. In this section, we will discuss the contributions of oligodendrocytes to polyQ SCAs across human, animal, and in vitro studies.

### 2.1. White Matter Abnormalities Are Observed in Patients with SCA1, 2, 3, and 7

Noninvasive imaging techniques have been explored in polyQ SCA patients to define white matter microstructure and neurochemical changes in disease progression (Figure 1B and Table 1). Most of these studies use diffusion tensor imaging (DTI), a method to visualize the microstructure of white matter tracts [32,33,34]. As white matter tracts consist of bundles of myelinated axons, DTI is an elegant method to noninvasively observe myelination index changes in human patients. Starting with SCA1, Park et al. (2020) assessed white matter changes via DTI in both symptomatic SCA1 patients and patients who were carriers of the *ATXN1* mutation but were not yet exhibiting ataxia symptoms (“preataxic”) [32]. In symptomatic patients compared to healthy controls, the middle cerebellar peduncle had significantly increased axial diffusivity [32]. Another study sought to determine whether there were correlations between SCA1 disease severity (as measured by the Scale for Assessment and Rating of Ataxia (SARA) score), duration of disease, and DTI metrics [35]. Interestingly, fractional anisotropy values in the superior cerebellar peduncle had a direct linear relationship with the duration of illness and an indirect linear relationship with the SARA score [35]. This finding suggests there are more progressive white matter disruptions in patients with severe ataxia, namely, those who have high SARA scores and less time from diagnosis until death (i.e., short disease duration) [35]. More recently, authors found that white matter damage occurs prior to gray matter damage in SCA1 and 3 [36]. In SCA1, neurochemical MRI metrics, including total creatine (tCr) and total *N*-acetylaspartate (tNAA) in the pons, were highly sensitive to preataxic changes, with higher tCr and lower tNAA being observed in preataxic SCA1 patients compared to controls [36]. Higher tCr indicates energy metabolism deficits and a lower tNAA to tCr ratio indicates reduced neurological status [36]. These findings implicate early white matter microstructural and neurochemical changes in the progression of SCA1.

White matter microstructural changes were similarly observed in the hemispheres, vermis, and peduncles of the cerebellum as well as in the pons of symptomatic SCA2 patients compared to controls [37]. More recently, lower axial diffusivity in the right corticospinal tract and right superior cerebellar peduncle, two regions vulnerable to neuron loss in SCA2, was associated with a higher SARA score [38]. Similar to SCA1 neurochemical changes, a significant reduction in the NAA to Cr ratio was observed in the cerebellar hemisphere of SCA2 patients compared to controls [39]. As previously mentioned, myelin contains a high proportion of lipids, including galactosylceramide and sulfatide, meaning lipid dysregulation could partially explain white matte microstructural abnormalities. Indeed, one study found significantly decreased sulfatide and galactosylceramide in postmortem cerebellar tissue of an SCA2 patient compared to two healthy controls [40]. In a previous study, this same patient sample was reported to have severe cranial nerve demyelination [58]. These studies demonstrate that white matter microstructural changes in subregions of the cerebellum are related to disease severity in SCA1 and 2 and that neurochemical abnormalities are also shared between these two diseases.

Significant changes in white matter have been observed in disease-vulnerable brain regions of SCA3 patients, including the pons, cerebellar peduncles, dentate nucleus, thalamus, and midbrain [41,42]. In the cerebellar lobules, reduced myelin staining and myelin basic protein has been discovered in end-stage postmortem cerebellar tissue from SCA3 patients compared to controls [49,50]. One DTI study in the pons of SCA3 patients found a direct relationship between fractional anisotropy values and disease duration [59]. Finally, a more recent paper demonstrated that changes to motor network white matter precede ataxia symptoms and are associated with disease severity [43]. Therefore, white matter microstructural alterations in SCA3 occur early [36] and progress in line with the expected regional neuronal degeneration in this disease [60], emphasizing the utility of DTI metrics in detecting changes in preataxic patients. As for neurochemical changes, decreased tNAA has been reported in disease-vulnerable brain regions of SCA3 patients, including the cerebellar white matter and vermis [44], with tNAA levels inversely relating to disease severity [45]. The ratio of NAA to Cr was also found to be decreased in cerebellar hemispheres, the dentate nucleus, and the vermis of SCA3 patients [46,47,48]. In sum, neurochemical abnormalities and white matter microstructural changes worsen progressively across SCA3 disease.

Patients with SCA7 share the white matter abnormalities found in patients with SCA1, 2, and 3. Loss of myelinated axons is well-supported throughout the brain of SCA7 patients, including the cerebellar white matter, corpus callosum, oculomotor nerve, and spinocerebellar tracts [52,53]. Afferent and efferent projections in the cerebellar peduncles appear to be affected on a microstructural level, with this area having decreased fractional anisotropy and increased mean diffusivity in SCA7 patients [54]. Supporting this, a significant association between mean diffusivity and SARA score was established in the middle occipital gyrus, superior cerebellar peduncles, and anterior cerebellar white matter [54]. Such observations in the cerebellum are in line with what would be expected given that this is a disease-vulnerable brain region with neuropathological abnormalities [52,53]. More recently, one study used an innovative approach to account for one of the limitations of DTI that relates to changes in CSF-like free water [55]. Given the significant brain atrophy in neurodegenerative diseases, SCA7 included, CSF-like free water can increase, which confounds DTI measures relying on diffusion of water molecules [55,61]. To overcome this barrier, Parker and colleagues estimated DTI metrics in the parenchyma only, excluding data from the CSF-like free water compartment, a method with higher test-retest reliability than standard DTI analysis [62]. The authors found microstructural abnormalities (increased mean diffusivity and decreased fractional anisotropy) throughout the entire brain parenchyma of SCA7 patients, including white matter tracts previously implicated in disease, such as the cerebellar peduncles and corticospinal tract [55]. SCA7 cerebellar parenchymal tissue volume and whole-brain parenchymal fractional anisotropy were inversely correlated with SARA score. These findings suggest progressive white matter impairments in SCA7 patients. In agreement with the DTI evidence, myelin staining of one late-onset female SCA7 patient reveals myelin pallor and/or atrophy in a large number of fiber tracts within the central nervous system outside of the optic tracts [56]. These findings are supported by another case study of an adult-onset male SCA7 patient [57] who had a larger CAG repeat expansion, earlier onset of disease, and shorter disease duration than the aforementioned female patient. In the future, the mechanism of these impairments in white matter microstructure could be elucidated using animal models of SCA7.

In contrast to the clarity of evidence supporting oligodendrocyte involvement in SCA1, 2, 3, and 7, there is inconsistent evidence of white matter changes in SCA6 and 17. To detail SCA6 first, one study comparing nine SCA6 patients to 15 healthy controls found no significant decreases in white matter in the brainstem, pons, and cerebellar peduncles, although alterations in white matter were observed in SCA3 patients in the same study [41]. However, there is still some indirect evidence supporting continued investigation of oligodendrocyte signatures in this disease. For example, minimal but significant white matter microstructural damage was discovered in eight SCA6 patient brains, along with volume loss in disease-vulnerable brain regions [32]. In agreement, one study of 14 SCA6 patients surprisingly found alterations in DTI metrics in SCA6 [51]. The superior cerebellar peduncle had the largest difference in DTI metrics between SCA6 patients and controls, with a decrease in radial diffusivity and increase in fractional anisotropy in preataxic patients and an increase in radial diffusivity and decrease in fractional anisotropy in moderate and severe symptomatic patients. The authors conclude that given the decrease in superior cerebellar peduncle volume and number of streamlines, the axons of SCA6 patients could be thinner than controls. In turn, thinner axons could restrict water flow, leading to a change in DTI metrics. This study suggests that thinner axons in the disease-vulnerable area of the superior cerebellar peduncle are a signature of SCA6, but this does not directly implicate oligodendrocytes. The next question would be how myelination is changed concomitant with changes in axon thickness; future studies should assess g-ratio and axon caliber using transmission electron microscopy of human postmortem samples or SCA6 mouse models to more precisely determine whether oligodendrocytes are changed.

As for SCA17, several studies demonstrate a deficit in bioenergetics that could indirectly contribute to oligodendrocyte impairments. Positron emission tomography (PET) scans of SCA17 preataxic and symptomatic patients reveal decreased metabolism of glucose in the striatum, cuneus, cingulum, and parietal lobe [63]. While decreased glucose metabolism could affect a number of processes, it is well established that oligodendrocytes have an immense demand for energy. Indeed, hypoglycemia inhibits OPC differentiation and migration in vitro [64,65], suggesting that glucose metabolism deficits could affect neurons and glia alike. This calls for additional in vitro and in vivo research on how decreased glucose metabolism could affect oligodendrocytes and the functional ramifications of such a change.

Together, these studies highlight the utility of using DTI metrics as a biomarker of SCA1, 2, 3, and 7 disease severity. There is extensive support for white matter changes in these diseases from cross-sectional studies that could be strengthened by future studies incorporating longitudinal assessments. Additional research will be needed to determine if the same is true for SCA6 and 17. As demonstrated by Park et al. (2020), this noninvasive technology has the potential to detect white matter microstructural abnormalities before the onset of severe ataxia, meaning that there may be a window of opportunity for therapeutic intervention. Developing such therapeutic solutions requires the use of animal models to probe the underlying cellular and molecular mechanisms of white matter changes.

### 2.2. Oligodendrocyte Dysregulation Is Observed in SCA1 and 3 Rodent Models

Recent RNAseq studies of SCA1 and 3 mouse models reveal perturbed oligodendrocyte maturation in disease (Table 2). For SCA1, authors conducted single-cell RNAseq on the cerebellum from heterozygous knock-in *Atxn1*^154^^*Q/+*^ mice and human SCA1 patients [66] (Figure 1C). In human and mouse samples, they found dysregulation of OPC and oligodendrocyte genes [66]. Specifically, many downregulated genes corresponded to transcripts found in mature oligodendrocytes [66]. This finding prompted the authors to further probe oligodendrocyte maturation in the human dataset. Using a strategy that allowed them to determine the path cells took to mature to their final state, the authors found that the OPCs from control samples followed one of two courses to become oligodendrocytes [66]. In contrast, OPCs from SCA1 patients followed a single course to mature into oligodendrocytes [66]. This means that SCA1 patients lacked a subset of maturing oligodendrocytes [66]. Compounding this, OPCs that did reach the mature state were still different from the mature oligodendrocytes of control samples as they had lower expression of mature transcripts, including the myelin transcripts *MOBP*, *MBP*, and *MOG* [66] (Figure 1A). These findings suggest perturbations in oligodendrocyte maturation in both human SCA1 patients and *Atxn1*^154^^*Q/+*^ knock-in mice.

To our knowledge, there are no studies directly addressing oligodendrocyte maturation in SCA2. However, several studies support deficits in the pathways involved in lipid synthesis, suggesting the involvement of oligodendrocytes in disease (Table 2). One study used the *Atxn2*-CAG100 knock-in mouse model and evaluated transcript levels in the spinal cord at two timepoints: 10 weeks and 14 months [67] (Figure 1C). Assessing preataxic (10 weeks) and symptomatic (14 months) timepoints allowed the authors to identify transcripts that became more dysregulated with disease progression [67]. Many of the transcripts identified were related to myelin and lipid synthesis, including *Dhcr24*, *Cyp51a1*, *Cyp36a1*, and *Msmo1*, among others. All of these genes are involved in the cholesterol biosynthesis pathway, suggesting vast dysregulation of this process in SCA2. In relation to oligodendrocytes, the authors reported downregulation of *Nat8l*, which codes for *N*-acetyltransferase 8-like [67], the neuronal enzyme that synthesizes N-acetylaspartate (NAA) by combining acetyl-CoA and aspartate [71]. Downregulation of *Nat8l*, and therefore NAA, affects oligodendrocytes because NAA is shuttled out of neurons and into oligodendrocytes, where it gets broken down and integrated into lipids [71,72]. Recall that myelin is composed of approximately 75–80% lipids, meaning that disruptions in lipid synthesis can affect myelin [23,72]. Indeed, there is broad support in the literature for reduced myelin synthesis due to decreases in NAA [72,73]. In summary, *Nat8l* expression progressively decreases across the course of SCA2, affecting lipid and myelin synthesis [67]. In support of this finding, two other papers from this lab, which generated the *Atxn2*-CAG100 knock-in mouse model, demonstrated (1) progressive reductions of Nat8l protein in the cerebellum, extending the aforementioned findings in the spinal cord to this disease-vulnerable area [68], and (2) dysregulation of various myelin-enriched lipids in the cerebellum and spinal cord [40]. These articles emphasize the utility of defects in lipid synthesis as a possible biomarker for disease progression. Tangentially related, an intermediate number of *ATXN2* CAG repeats has long been known to be a risk factor for ALS [74,75]. New evidence indicates a decrease in oligodendrocytes and motor neurons in ALS patient postmortem spinal cord samples via RNAseq [15]. As changes in oligodendrocyte number have not been investigated in SCA2 to our knowledge, this represents an exciting advance connecting *ATXN2* as a risk gene and oligodendrocyte dysregulation in ALS, although more direct evidence is required.

The finding of irregular oligodendrocyte maturation is not unique to SCA1 as our lab recently found the same disruption in SCA3 [50,69,70] (Table 2). Analysis of bulk RNAseq data from transgenic SCA3 mice overexpressing human *ATXN3* with 84 CAG repeats (YACQ84) revealed impaired oligodendrocyte maturation in the disease-vulnerable brain regions of the pons, deep cerebellar nuclei, brainstem, and corticospinal tract relative to wild-type controls [69]. Expanding on this, we found increased expression of OPC transcripts (*Smoc1*) and decreased expression of mature oligodendrocyte transcripts (*Mobp* and *Mal*) in the brainstem of YACQ84 mice at a symptomatic timepoint [69]. Mature oligodendrocyte signatures are also perturbed in the cerebellum and pons when *Atxn3* is expressed at endogenous levels [76,77]. For example, in the cerebellum of 12-month-old hemizygote knock-in mice (WT/304Q), myelin basic protein expressed in mature oligodendrocytes was significantly decreased relative to wild-type littermates, suggesting impairment of mature oligodendrocytes in an endogenous expression system [77]. In an effort to understand whether the oligodendrocyte maturation deficit in SCA3 was cell autonomous, our lab conducted primary oligodendrocyte cell culture. We isolated OPCs from P5–7 YACQ84 mice, a timepoint chosen to isolate proliferating OPCs rather than any differentiating cells, as the highest rate of myelination in mice occurs between P7 and 21 [78]. Remarkably, we found the maturation deficit to be recapitulated, with greater number of OPCs/immature OLs and fewer mature OLs in SCA3 compared to WT [69,70] (Figure 1A). The next question was whether the maturation deficit was due to a gain-of-function mechanism. We found that oligodendrocytes isolated from *Atxn3* knock-out mice matured normally, confirming the toxic gain-of-function mechanism [70]. These findings are valuable for two reasons. First, they emphasize a shared impairment of oligodendrocyte maturation in SCA1 and 3. Second, the observation that mutant ATXN3 acts by a toxic gain-of-function mechanism provides insight into how we could intervene by reducing levels of the mutant protein. As oligodendrocytes are a common target in SCA1 and 3, this cell type represents a promising therapeutic target.

As for SCA6, mechanistic evidence suggests a link between the disease hallmark of Purkinje cell torpedoes and myelination. Torpedoes are focal swellings or spheroids of an axon and are thought to increase in number on Purkinje cells as a response to cerebellar injury [79,80,81]. Increased torpedo formation has been demonstrated in essential tremor [82], a cerebellum-linked condition, as well as SCA6 [83]. One study evaluated the number of torpedoes on Purkinje cells in transgenic SCA6^84Q/84Q^ mice [80]. This study found the highest number of torpedoes at P11, most of which exist on myelinated axons. This is intriguing because the highest rate of myelination in mice occurs between P7 and 21 [78], possibly suggesting a connection between the development of the disease signature of torpedoes and axonal myelination. Most evidence in SCA6 literature supports minimal involvement of oligodendrocytes in disease pathology. However, as mentioned in Section 2.1, a few studies have demonstrated changes in white matter and axon caliber in patients with SCA6 [32,51]. Additional research could yield a more complete answer about whether oligodendrocytes contribute to SCA6 pathogenesis.

Like SCA6, there are few studies assessing white matter alterations in SCA7 and 17 rodent models. Only one study in SCA17 TBPQ64 rats applied DTI and found a decrease in fractional anisotropy in the external capsule, suggesting decreases in myelin sheath integrity surrounding axons projecting to the striatum [84]. In SCA7, one study conducted a neuroanatomical study of end-stage SCA7 knock-in mice (140Q/5Q) and found morphological alterations to the white matter of the corpus callosum, hippocampus, anterior commissure, and internal capsule [85]. Given the wealth of evidence for white matter alterations in human SCA7 patients, the dearth of evidence using animal models emphasizes an area ripe for further investigation.

In sum, white matter microstructural changes progressing in preataxic to symptomatic patients point toward the involvement of oligodendrocytes early in SCA1, 2, 3, and 7 disease. Importantly, mouse models in some of these diseases recapitulate oligodendrocyte impairments, emphasizing their utility as a preclinical model. Moving forward, it will be invaluable to characterize the basic biology of oligodendrocytes as we know little about how they handle misfolded proteins, which are a hallmark of neurodegenerative diseases [70]. After all, it is imperative first to understand how oligodendrocytes function under normal conditions before investigating how they change with disease.

## 3. Peripheral Nervous System Myelinating Glia: Schwann Cells

**Figure 2:**
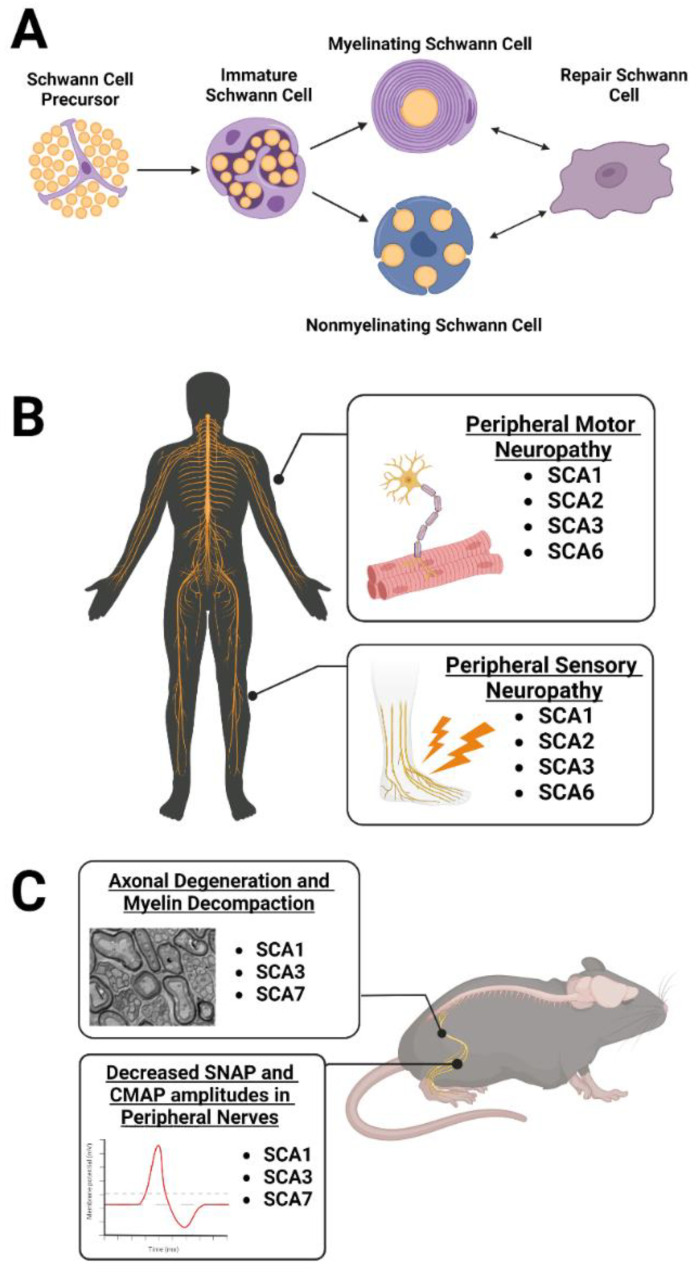
Evidence for peripheral neuropathy and Schwann cell involvement in polyQ SCA literature. (**A**) Schematic of Schwann cell maturation. (**B**) SCA1, 2, 3, and 6 patients are known to present with chronic pain related to peripheral sensory neuropathy as well as reductions in compound muscle action potentials and sensory nerve action potentials that relate to peripheral motor neuropathy. (**C**) Mouse studies assessing features of polyQ SCA disease-related peripheral neuropathy report axonal degeneration, myelin decompaction, and decreased sensory nerve action potential (SNAP) and compound muscle action potentials (CMAP) amplitudes in peripheral nerves, most noticeably in SCA1, 3, and 7. Figure created using BioRender.

The peripheral nervous system has a remarkable ability to regenerate itself after axonal injury/loss through mechanisms and pathways not entirely shared with the central nervous system. Differences in regenerative capacity between the central nervous system and peripheral nervous system are due to the dynamic repair capabilities of the Schwann cell, the myelinating glial cell of the peripheral nervous system [86]. There are two main Schwann cell subtypes relating to the function and number of axons ensheathed by a single Schwann cell (Figure 2A). The first subtype is the myelinating Schwann cell, which myelinate large caliber (greater than 1 µm in diameter) axons in a 1:1 ratio, with an individual Schwann cell engulfing one distinct section of the axon to generate the myelin sheath [86]. Consequently, a large population of Schwann cells are required to ensheath the complete length of an axon, enabling rapid saltatory impulse conduction for precise motor function. The second subtype consists of nonmyelinating or Remak Schwann cells. These engulf numerous small caliber axons without myelinating them and are typically associated with sensory information [86].

Dysfunction in peripheral white matter, such as that seen in hereditary ataxias, threatens critical Schwann cell–axon interactions and results in various neurological deficits [16]. In the context of peripheral nerve injury or degeneration, myelinating Schwann cells demyelinate their respective axons and, along with nonmyelinating Schwann cells, undergo extensive transcriptional reprogramming to activate a distinct “repair” phenotype [86]. Within this phenotype, Schwann cells coordinate critical functions for remyelination of axons and subsequent nerve regeneration. In a process known as Wallerian degeneration, Schwann cells aid in the disintegration of distal injured axons, clearing axon and myelin debris through autophagy, myelinophagy, and recruitment of macrophages [87]. Simultaneously, Schwann cell secretion of trophic factors fosters the survival of injured neurons and aids axonal regeneration [88]. Repair Schwann cells also extend long processes designed to guide the regenerating axon’s growth toward its previous target. At the end of these processes, Schwann cells upregulate promyelinating genes and once again ensheath the axon to remyelinate the regenerated neuron [86]. This phenomenon is unique to the peripheral nervous system and provides the opportunity to harness such regenerative capacity for therapeutic benefit in neurodegenerative disease.

### 3.1. Peripheral Neuropathy Is Observed in Patients with SCA1, 2, 3, and 6

Despite the striking prevalence of peripheral nerve pathology in hereditary ataxias, the role of peripheral cells in these diseases remains understudied. In the general population, 8.6% of nondiabetic human subjects aged 40–69 and 25.4% of those aged 70 and older were diagnosed with peripheral neuropathy via monofilament testing in a sample consisting of 5200 participants [89]. Narrowing the sample to patients with hereditary ataxia, a study of 162 patients revealed electrophysiologically defined peripheral neuropathy in 82% of SCA1, 63% of SCA2, 55% of SCA3, and 22% of SCA6 patients [18] (Figure 2B). One salient feature of ataxia-related peripheral neuropathy is velocity reduction in sensory nerve action potential (SNAP), compound muscle action potential (CMAP), and sensorimotor nerve conduction. Decreases in these velocities are present in SCA1, 2, 3, and 6 patients [18], with sensory and motor neuropathy being most commonly found in SCA2 patients. The process of axonal degeneration in SCA2 is suggested to begin specifically at the neuronal body rather than at distal axon sites [90], in contrast to peripheral nerve injury models, where degeneration is induced along the axon length [87]. Interestingly, SCA2 clinical peripheral neuropathy features may begin before central nervous system disease onset. Sensory nerve latencies and amplitudes in peripheral nerves worsened prior to clinically defined onset of central nervous system deterioration in SCA2 patients evaluated over a 20-year period, suggesting peripheral symptoms could stand as predictive biomarkers for diagnosis [17]. Peripheral neuropathy as a central nervous system independent symptom is shared in some instances of SCA3 as well. Four patients with intermediate CAG expansions in *ATXN3* were reported to have decreased CMAPs, SNAPs, and nerve conduction velocities consistent with motor neuron loss and sensory neuropathy after onset of limb weakness and muscle atrophy [91]. However, central nervous system related ataxia was absent in all cases, highlighting the periphery’s autonomy in this disease context.

### 3.2. Schwann Cell Dysregulation Is Observed in SCA1, 3, and 7 Rodent Models

Toward the underlying cause of these changes, Schwann cells were directly implicated in polyQ SCA pathology in studies assessing nerve conduction, peripheral myelination, and cellular disease protein accumulation in animal models (Figure 2C). One study investigated spinal motor neurons in SCA1 knock-in mice with 154 polyQ repeats aged beyond 25 weeks, finding thinner myelin sheaths and demyelination on these axons using MBP staining [92]. As spinal motor neurons send information to the muscles, the authors questioned whether thinning myelin would affect muscle responses and found lower CMAP amplitudes as well as longer CMAP latency and duration in diseased mice compared to controls [92]. This suggests that axonal neuropathy affects the spinal motor neurons of SCA1 knock-in mice. In a follow-up study to evaluate whether axons or myelin degenerate first in SCA1 knock-in mice, the same lab found smaller axons in one-month-old SCA1 mice compared to controls, indicating axonal degeneration prior to symptom onset in these mice [93]. Myelin thickness was not affected at this timepoint but was decreased at three months of age in diseased mice relative to controls, suggesting that myelin thinning is secondary to axonal degeneration in this mouse model [93]. In SCA3 patients, the authors found that reduced SNAPs and nerve conduction velocities were coupled with decreased myelin thickness in the sural nerve, marked by increased g-ratios (ratio of inner-to-outer diameter of the myelinated axon) [16]. Histological visualization also revealed nuclear ATXN3 accumulation in dorsal root ganglion and anterior horn motor neuron nuclei, along with ATXN3 accumulation in Schwann cell cytoplasm, tying these cells to a pathological hallmark of SCA3 disease [16]. Another study used a YAC transgenic mouse expressing *ATXN3* with expanded CAG repeats to assess the effect of *ATXN3* CAG repeat length on sciatic nerve myelination. The authors found significant demyelination abnormalities at 6, 9, and 12 months of age in ATXN3^67.2/84.2^ double-transgenic mice followed by severe myelin abnormalities and incidence of thinly myelinated axons by 14 months of age [94]. In contrast, mice with only 15.4 CAG repeats in the *ATXN3* gene construct displayed comparable myelination to nontransgenic mice [94]. These studies establish a link between exacerbated polyQ repeat expression and an abnormal peripheral nerve phenotype specifically in SCA3. Finally, studies of SCA7-related nerve pathology found that SCA7^140Q/5Q^ knock-in mice had reduced motor M-wave and sensory H-wave amplitudes in the sciatic nerve relative to WT mice as recorded by electromyography. These findings were paired with morphological abnormalities in mutant mice relative to WT, such as myelin degeneration markers, infolding-like structures, abnormal Remak bundles, and higher incidence of autophagy [85]. In summary, these studies emphasize the need for therapeutics that target the periphery in addition to the brain. Such therapeutics are particularly necessary as peripheral neuropathy causes lifelong disabilities despite the regenerative ability of Schwann cells. Additional research is needed in this field as current treatments, including autologous nerve grafting, have been inadequate, with only 25% of patients regaining full motor function and 3% recovering sensory function [86]. Moreover, chronic pain poses an immense challenge for ataxia patients as 33% of SCA3 patients report severe chronic pain, particularly in the lower back and lower extremities [95]. More intense pain is linked to longer CAG repeat expansions, meaning certain patients experience higher levels of distress and as such require more efficacious treatments targeting the peripheral nervous system to restore quality of life.

## 4. Therapeutic Considerations for Targeting Myelinating Glia

### 4.1. Targeting Oligodendrocytes

As there are no disease-modifying or curative treatments for SCAs, preclinical research using mouse models to discover disease modifiers is of utmost importance. Antisense oligonucleotides (ASOs) are a well-established gene-suppression method that consist of a single-stranded oligonucleotide binding target mRNA to block translation. Preclinical ASO studies in SCA1, 2, and 3 have reported great success in rescuing mouse model motor and pathological phenotypes [96,97,98,99]. In SCA2 and 3 ASO studies, electrophysiological phenotypes were also rescued [96]. Consequently, an ongoing clinical trial in SCA3 patients is testing the safety and efficacy of an anti-*ATXN3* ASO (ClinicalTrials.gov identifier: NCT05160558, accessed on 20 January 2023), while another is testing the safety and efficacy of an anti-*ATXN2* ASO in ALS patients (ClinicalTrials.gov identifier: NCT04494256, accessed on 20 January 2023). Toward therapeutic considerations for targeting myelinating glia, our lab recently demonstrated that anti-*ATXN3* ASOs in an SCA3 mouse model rescue the deficit in oligodendrocyte maturation when administered to symptomatic YACQ84 mice at eight weeks of age [100]. Intriguingly, when we determined how myelination of various central nervous system white matter tracts was affected by the ASO, we found hypermyelination of the corpus callosum, an area that had normal oligodendrocyte maturation [100]. This suggests that determining what prevents oligodendrocyte impairments in disease-protected brain regions is as interesting as what causes oligodendrocyte impairments in disease-vulnerable brain regions. Investigating brain regions that do not display neuronal loss or oligodendrocyte changes may prove fruitful across polyQ SCAs. Additional indirect support of the therapeutic potential of targeting oligodendrocytes comes from a paper that found overexpressing CYP46A1, an enzyme involved in cholesterol turnover in the brain and cholesterol efflux from the brain, rescued motor phenotypes in a transgenic SCA3 mouse model overexpressing N-terminally truncated human *ATXN3* with 69 repeats only in Purkinje cells [101]. This again demonstrates the importance of targeting processes related to oligodendrocytes for therapeutic benefit as myelin is cholesterol-rich [102], accounting for up to 80% of cholesterol in the brain [103,104,105].

Another exciting opportunity comes from using stem cells for drug screens. While primary oligodendrocyte cell culture from mouse models of disease is useful in determining underlying cellular and molecular mechanisms, this is not a perfect representation of human disease. There are important differences between human and rodent oligodendrocytes that may be relevant in studies aiming to restore oligodendrocyte maturation and myelination [106]. For example, the length of time devoted to oligodendrocyte generation and myelination are vastly different between humans and rodents [107]. Therefore, when testing therapeutics to improve oligodendrocyte maturation and myelination, it is essential to maintain human relevance while still making mechanistic advances. Generation of oligodendrocytes from human-induced pluripotent stem cells (iPSCs) or embryonic stem cells (ESCs) represents an ideal balance of a human-relevant model for mechanistic study. Original protocols differentiated iPSCs into induced oligodendrocytes (iOLs) using growth factors that took 70–150 days for iOLs to reach a mature myelinating state [108,109,110]. To improve timing, recent proof-of-concept papers highlight the potential for master transcription factors (TFs) to reprogram iPSCs into iOLs at a much faster rate [108,111,112], which is standard practice for iNeuron reprogramming. Therefore, stem cells could provide an efficient and standardized tool for the generation of human iOLs from an expandable source.

Another polyQ disease, Huntington’s disease (HD), has led the way in investigations of oligodendrocyte contributions to pathophysiology, specifically finding a cell-autonomous defect in oligodendrocyte differentiation [13,113]. One study extended this finding to understand how CD140a-expressing OPCs derived from mutant huntingtin (mHTT) hESCs differ from OPCs derived from control hESCs [114]. Through RNAseq on mHTT and control OPCs, they found that gene ontology biological processes affected in disease were glial cell differentiation and myelination, axon guidance and axonogenesis, and regulation of synapse structure and synaptic signaling. The first module included significant downregulation of mature oligodendrocyte myelin-related protein transcripts, including *Mbp*, *Mag*, *Omg*, *Plp1*, and *Mog*. The generation of human mHTT OPCs represents a significant advancement and an opportunity to use these cells to test various therapeutics. For example, overexpressing SOX10 and MYRF significantly increased oligodendrocyte differentiation and myelin gene expression in mHTT OPCs in vitro [114], suggesting that therapeutics targeting oligodendrocyte differentiation should be further evaluated across all polyQ diseases.

### 4.2. Targeting Schwann Cells

To date, there are limited studies of Schwann cells as a therapeutic target for polyQ-SCA-related peripheral neuropathy. It is known that after nerve injury, Schwann cells dedifferentiate and undergo transcriptional reprogramming to facilitate axonal regeneration [86]. As was previously discussed, axonal damage and demyelination are persistent features of peripheral neuropathy in SCA patients. Thus, targeting Schwann cell support of peripheral nerves may have therapeutic value. A critical function of repair-phenotype Schwann cells is to release trophic factors to foster successful axonal regeneration [88]. In cell culture, it is known that iPSCs can differentiate into Schwann cells and release neurotrophic factors, such as brain-derived neurotrophic factor, glial-cell-derived neurotrophic factor, nerve growth factor, and neurotrophin-3, as well as extracellular matrix factors to stimulate axonal growth similarly to Schwann cells in vivo [88]. It is therefore possible for iPSCs to be harnessed to manufacture Schwann cells with varied expression of trophic factors to attenuate peripheral neuropathy [115]. One protocol successfully produced self-renewing Schwann cell precursor cells from human pluripotent stem cells (hPSCs) using TGF-beta, glycogen synthase kinase-3 inhibitors, and neuregulin-1. These cells matured into functional Schwann cells within one week, secreting the previously mentioned factors and capably myelinating rat DRG axons in vitro [116]. However, this model displayed limitations in myelinating efficiency, which may be attributable to species differences or cell–axon ratios. It still remains unknown how to optimize differentiation protocols to generate fully functionable Schwann cells in vitro. Once accomplished, however, the axonal degeneration model Schwann cells could provide a valuable platform for therapeutic testing of drugs targeting peripheral symptoms in hereditary ataxia.

To further capitalize on the advantages of cell culture, the delineation of biomarkers for the repair phenotype of Schwann cells could open the door to developing autologous Schwann cell transplant in degenerating peripheral nerves. Specifically, skin Schwann cell cultures have shown promise and could potentially allow for monitoring of the clonal response of Schwann cells to injury using Cre reporters and genetic barcoding [117,118]. To identify dysfunctional pathways and biomarkers in ataxia-related Schwann cell degeneration, genetic techniques, such as single-cell RNAseq, as well as knock-down of remyelination “gatekeeper” genes via a transgenic model, miRNAs, siRNAs, or ASOs would provide valuable insight into the mechanistic underpinnings of peripheral neuropathy in these diseases [119]. Upon identification of dysfunctional pathways in each disease, Schwann cell phenotypes of interest can be run through small molecule/drug screens to investigate possible novel therapeutics. In fact, RNA interference techniques have shown to effectively ameliorate peripheral neuropathy-related pathology induced by toxic gain-of-function disease proteins. Weekly subcutaneous ASO injections dose-dependently reduced PMP22 mRNA transcript levels and improved functional and ultrastructural markers in a Charcot–Marie–Tooth (CMT) mouse model characterized by robust peripheral neuropathy [120]. Similarly, the CMT1A mouse model underwent efficient Schwann cell transduction of an AAV-packaged miRNA targeting overexpressed PMP22 mRNA transcripts. Improvements in peripheral nerve function and myelination in the lumbar roots and femoral motor nerves coupled AAV transduction, solidifying proof of principle for the translatable value of a gene therapy approach toward peripheral neuropathy [121].

## 5. Conclusions

Disease-associated glial signatures are rapidly emerging as important contributors to polyQ SCA pathophysiology in the central and peripheral nervous system. Further research on the basic biology of oligodendrocytes and Schwann cells, including how they handle the neurodegenerative disease hallmark of misfolded and/or aggregated proteins, is necessary to fully understand how these cells respond to disease. Such research has the potential to broadly impact the neurodegenerative disease field that shares the theme of disease-associated myelinating cell signatures. Importantly, as polyQ SCAs have well-described monogenic etiology, these diseases can serve as paradigms for further research across the neurodegenerative disease field of glial contributions to disease and therapeutic opportunities.

## Figures and Tables

**Table 1:** Evidence for white matter alterations in patients with polyQ SCAs.

Disease	Method	Findings *	Ref
SCA1	DTI	Increased axial diffusivity in the middle cerebellar peduncle	[32]
		Fractional anisotropy values in the superior cerebellar peduncle are positively correlated with duration of illness and negatively correlated with SARA score	[35]
	MRS	Higher tCr and lower tNAA in preataxic patients	[36]
SCA2	DTI	White matter microstructural changes in the pons and cerebellar peduncles, hemispheres, and vermis	[37]
		Axial diffusivity in the right corticospinal tract and right superior cerebellar peduncle is negatively correlated with SARA scores	[38]
	MRS	Decreased NAA to Cr ratio in the cerebellar hemisphere	[39]
	Other	Reduced sulfatide and galactosylceramide in one postmortem patient cerebellum	[40]
SCA3	DTI	White matter alterations in disease-vulnerable brain regions (cerebellar peduncles, dentate nucleus, pons, midbrain, and thalamus)	[41,42]
		Direct relationship between disease duration and fractional anisotropy values	[43]
		Motor network white matter changes correlate with disease severity and occur prior to onset of ataxia symptoms	[43]
	MRS	Reduced tNAA in cerebellar vermis and white matter	[44]
		Inverse relationship between tNAA levels and disease severity	[45]
		Reduced NAA to Cr ratio in cerebellar vermis, hemispheres, and dentate nucleus	[46,47,48]
	Other	Decreased myelin basic protein and myelin staining in patient postmortem cerebellar tissue	[49,50]
SCA6	DTI	Limited but significant damage to white matter microstructure	[32]
		Increased fractional anisotropy and decreased radial diffusivity and in the superior cerebellar peduncle of preataxic patients	[51]
		Decreased fractional anisotropy and increased radial diffusivity in the superior cerebellar peduncle of moderate to severe symptomatic patients	[51]
SCA7	DTI	DTI and MRI reveal loss of myelinated axons in the spinocerebellar tract, oculomotor nerve, cerebellar white matter, and corpus callosum	[52,53]
		Increased mean diffusivity and decreased fractional anisotropy of the cerebellar peduncles and corticospinal tract	[54,55]
		Significant correlation between mean diffusivity and SARA score in anterior cerebellar white matter, superior cerebellar peduncles, and middle occipital gyrus	[54]
		Inverse relationship between whole-brain parenchymal fractional anisotropy/cerebellar parenchymal tissue volume and SARA score	[55]
	Other	Myelin abnormalities in central nervous fiber tracts outside the optic tract in two adult-onset patients	[56,57]

* Relative to healthy control subjects. Abbreviations: CR, creatine; DTI, diffusion tensor imaging; MRI, magnetic resonance imaging; MRS, magnetic resonance spectroscopy; NAA, *N*-acetylaspartate; PET, positron emission tomography; SARA, Scale for Assessment and Rating of Ataxia; tCR, total creatine; tNAA, total *N*-acetylaspartate.

**Table 2:** Evidence for mature myelinating oligodendrocyte perturbations in polyQ SCA mouse models.

Disease	Mouse Model	Findings *	Ref
SCA1	Knock-in *Atxn1*^154Q/+^	Dysregulation of mature oligodendrocyte transcripts and protein in mouse cerebellar tissue. Cerebellar TEM analysis showed age-dependent reductions in myelination.	[66]
SCA2	*Atxn2*-CAG100 knock-in	Transcriptional dysregulation of myelin and lipid synthesis (including *Nat8l*) identified by RNAseq of mouse spinal cord. Decreased levels of cholesterol biosynthesis pathway intermediates identified by gas chromatography–mass spectrometry of mouse spinal cord.	[67]
		Decreased NAT8L in the cerebellum by Western blot analysis.	[68]
		Perturbations in the levels of myelin-enriched lipids in the cerebellum and spinal cord by liquid chromatography–mass spectrometry.	[40]
SCA3	YACQ84 transgenic	Decreased mature oligodendrocyte transcript and protein expression in the brainstem and cerebellum by RNAseq and Western blot analysis, respectively. This is paralleled by a reduced number of mature oligodendrocyte cell counts (immunohistochemistry) and thinner myelination (TEM analysis) in disease-vulnerable brain regions.	[69]
		Onset of mature oligodendrocyte transcriptional demise parallels onset of motor deficits.	[50]
		The maturation deficit is cell autonomous and due to a toxic gain of function by primary oligodendrocyte culture.	[69,70]
	*Atxn3* Q82 and Q300 knock-in	PolyQ repeat-dependent spatiotemporal dysregulation of mature oligodendrocyte transcripts; Q300 mice show dysfunction earlier than Q82 mice.	[50]

* Relative to wild-type control mice. Abbreviations: RNAseq, RNA sequencing.
